# Diagnostic yield of viral multiplex PCR during acute exacerbation of COPD admitted to the intensive care unit: a pilot study

**DOI:** 10.1038/s41598-024-51465-1

**Published:** 2024-01-11

**Authors:** Costa Salachas, Cherifa Gounane, Gaëtan Beduneau, Julien Lopinto, Matthieu Turpin, Corinne Amiel, Antoine Cuvelier, Marie Gueudin, Guillaume Voiriot, Muriel Fartoukh

**Affiliations:** 1 Assistance Publique – Hôpitaux de Paris, Service de Médecine Intensive Réanimation, Hôpital Tenon, 4, Rue de La Chine, 75020 Paris, France; 2 Département de Médecine Intensive Réanimation, Normandie Univ, UNIROUEN, UR 3830, CHU Rouen, 76000 Rouen, France; 3 Assistance Publique – Hôpitaux de Paris, Département de Virologie, Hôpital Tenon, 75020 Paris, France; 4 Normandie Univ, UNIROUEN, UR 3830, CHU Rouen, Service de Soins Intensifs Respiratoires, Rouen, France; 5 Département de Virologie, Normandie Univ, UNIROUEN, UNICAEN, UMR1311 INSERM DYNAMICURE, CHU Rouen, Rouen, France; 6 Sorbonne Université, Paris, France

**Keywords:** Diagnostic markers, Epidemiology, Viral infection

## Abstract

Acute exacerbation of chronic obstructive pulmonary disease (AECOPD) is one of the leading causes of admission to the intensive care unit, often triggered by a respiratory tract infection of bacterial or viral aetiology. Managing antibiotic therapy in this context remains a challenge. Respiratory panel molecular tests allow identifying viral aetiologies of AECOPD. We hypothesized that the systematic use of a respiratory multiplex PCR (mPCR) would help antibiotics saving in severe AECOPD. Our objectives were to describe the spectrum of infectious aetiologies of severe AECOPD, using a diagnostic approach combining conventional diagnostic tests and mPCR, and to measure antibiotics exposure. The study was bicentric, prospective, observational, and included 105 critically ill patients with a severe AECOPD of presumed infectious aetiology, in whom a respiratory mPCR with a viral panel was performed in addition to conventional microbiological tests. Altogether, the microbiological documentation rate was 50%, including bacteria alone (19%), respiratory viruses alone (16%), and mixed viruses and bacterial species (16%). The duration of antibiotic therapy was shorter in patients without documented bacterial infection (5.6 *vs*. 9 days; *P* = 0.0006). This pilot study suggests that molecular tests may help for the proper use of anti-infective treatments in critically ill patients with severe AECOPD.

## Introduction

Chronic obstructive pulmonary disease (COPD) is one of the leading cause of mortality and handicap, affecting more than 380 millions of people worldwide^1^. The natural course of COPD is marked by episodes of acute exacerbation (AECOPD), defined by an acute worsening of symptoms (mainly dyspnea, cough, sputum production). AECOPD is a common reason of admission to the intensive care unit (ICU)^1^, mainly caused by bacterial or viral infection^1^. The medical management of AECOPD is based on bronchodilators, steroids and antibiotics, the latter being often used in excess^1^. Controlling antimicrobial resistance is a major contemporary concern, and high priority objective for the World Health Organisation (WH0)^2^. Current international^1^ and French^3^ guidelines recommend a five-day course of antibiotics for patients with AECOPD and three cardinal Anthonisen’s symptoms (increased dyspnea, increased sputum volume, sputum purulence) or two of these symptoms if sputum purulence is one of them, or for patients who require mechanical ventilation. These guidelines, mostly based on clinical symptoms and history, may encourage liberal antibiotics use. Although of indisputable interest, conventional microbiological tests cultures provide delayed results, which do not allow rapid antibiotic streamlining.

Studies in the last decades have tested various strategies to limit antibiotics use in AECOPD. Firstly, several studies have evaluated strategies using biomarkers, like C-reactive protein (CRP)^4,5^ or procalcitonin (PCT)^6–9^. Unfortunately, their results are heterogeneous and conflicting, and thus guidelines^1,3^ do not recommend to generalize these biomarkers approaches, and suggest to run confirmatory trials with rigorous methodology. Secondly, by allowing rapid detection of viral pathogens, respiratory multiplex polymerase chain reaction (mPCR) may help avoid unnecessary antibiotics in viral AECOPD. Its contribution to antibiotics management and sparing has been suggested in acute lower respiratory tract infection (LRTI), but has not yet been confirmed^10,11^. Therefore, mPCR use in AECOPD is not currently mentioned in the guidelines^1,3^. Nevertheless, some data suggest a possible usefulness of this test for AECOPD in a subgroup of a randomized control trial^12^ and a retrospective cohort^13^. However, no study has tested the usefulness of mPCR in critically ill patients with AECOPD.

We hypothesized that improving the diagnosis of viral infection, using respiratory viral mPCR in patients admitted to the ICU for severe AECOPD, could be associated with a reduction in antibiotics utilization, without deleterious impact on the clinical outcomes of patients. The objectives of this pilot study were to describe the respiratory microorganisms identified during severe AECOPD, using conventional microbiological tests associated with respiratory viral mPCR, and to measure the overall duration of antibiotics exposure in the ICU.

## Patients and methods

The “EXAcerbations VIRales des patients ayant une BPCO” (EXAVIR) study was a prospective non-interventional bi-center cohort study conducted in the critical care units of Tenon Hospital, Paris, France, and Charles Nicolle Hospital, Rouen, France, two tertiary university-teaching hospitals. All adult patients (≥ 18 years old) with COPD admitted to the ICU or the affiliated step-down unit from December 2016 to August 2018 for AECOPD with a suspected infectious aetiology, and for whom a respiratory viral mPCR was obtained were eligible. Patients with acute pneumonia diagnosed at inclusion, exacerbations presumably caused by a left cardiac dysfunction, pregnant or lactating women, and patients deprived of liberty or under legal protection measure were not included.

AECOPD was diagnosed on clinical definition, according to the international recommendations^1^. COPD was established by respiratory function tests, or was highly suspected from the clinical history. Infectious aetiology was clinically suspected on clinical history and acute symptoms. The therapeutic management of AECOPD included bronchodilators, supplemental oxygen, and mechanical ventilation if needed, according to recommendations^1,3^.

Whenever possible, conventional microbiological investigation was performed before any antimicrobial treatment was administered, including respiratory tract specimen samples (sputum, tracheal aspirate or broncho alveolar lavage), blood cultures, and urinary antigen tests for *Legionella pneumophila* and *Streptococcus pneumoniae.* Empiric antimicrobial treatment was initiated according to the recommendations^1,3^ and to a known bronchial colonization if available. A nasopharyngeal swab was performed in all patients for respiratory mPCR, using either the FilmArray Respiratory Panel system (BioFire, Salt Lake City, UT) in Tenon hospital that includes 17 respiratory viruses (coronaviruses, adenovirus, human metapneumovirus, human enterovirus/ rhinovirus, respiratory syncytial virus, parainfluenza viruses and influenza viruses A and B) and three bacteria (*Chlamydophila pneumoniae, Mycoplasma pneumoniae,* and *Bordetella pertussis*)^14^, or the ePLEX Respiratory Panel system (GenMark DX) in Rouen hospital that includes 22 respiratory viruses (same viruses plus MERS coronavirus, more influenza strains and bocavirus) and four bacteria (same bacteria plus *Legionella pneumophila*)^15^. A bacterial aetiology of AECOPD (bacterial AECOPD) was diagnosed when a bacterial microorganism was identified in respiratory tract samples cultures, or blood cultures, or when urinary antigen tests were positive. Non-bacterial AECOPD was defined by the absence of bacterial documentation, regardless of any viral detection.

All methods were performed in accordance with the relevant guidelines and regulations.

### Statistical analysis

Continuous data were expressed as median (25th–75th percentiles) or mean (standard deviation) as appropriate, and were compared using the Student *t* test or a non-parametric test. Categorical variables, expressed as percentages, were compared using the Chi-square test or the Fisher’s exact test. Infectious aetiologies of AECOPD were categorised as viral, bacterial, or mixed (bacteria-viruses). Then, bacterial episodes of AECOPD (episodes with pure bacterial infection and episodes with mixed infection) and non-bacterial episodes of AECOPD (episodes with viral infection and episodes without documented infection) were compared. Statistical significance was defined as *P* values of less than 0.05. The objectives of this pilot study were to describe the respiratory microorganisms identified during severe AECOPD in patients admitted to the ICU and affiliated step-down unit, using conventional microbiological tests associated with respiratory viral mPCR, and to measure the overall duration of antibiotics exposure in the ICU and affiliated step-down unit, contrasting bacterial AECOPD and non-bacterial AECOPD. Based on a previous series involving a similar population of patients^9^, we estimated respective mean durations of antibiotics of 8 days and 5 days for bacterial and non-bacterial AECOPD (difference of 3 days ± 5 days). With a two-sided alpha risk of 5% and 80% power, 44 patients per group would be needed (88 in total). Anticipating a 10% rate of unusable data (due to lost to follow-up, or missing data for any reason), we aimed to include at least 100 patients. Data were analysed using the Stata software (V 13.1, College Station, TX).

### Ethical consideration

The study was approved by the relevant French authorities (Comité de Protection des Personnes-Est II, No 17/532; ANSM, No 2016-A01777-4), and written informed consent was obtained from all participants and/or their legal guardians.

## Results

**Figure 1:**
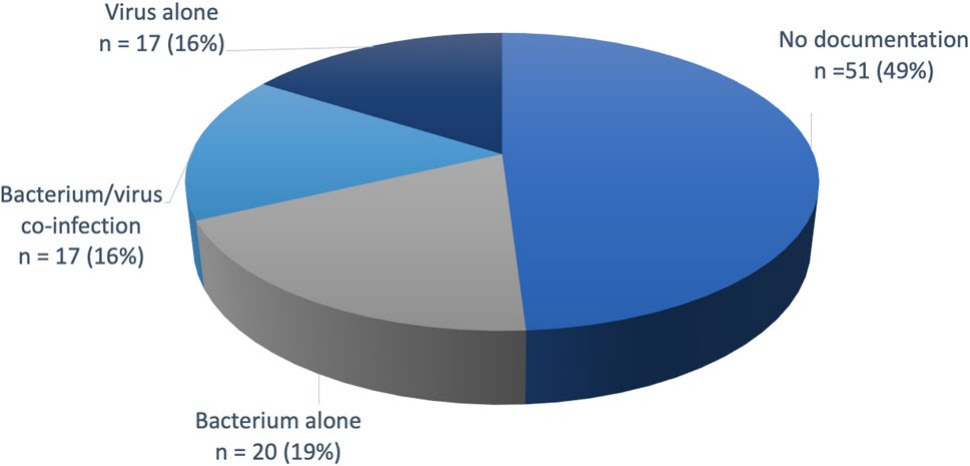
Microbial spectrum of severe AECOPD.

During the study period, 105 AECOPD patients with a suspected infectious aetiology were enrolled. Baseline characteristics are detailed in Table 1. Conventional respiratory investigations were performed in 75 patients (72%) (Table 2). Bacterial species were identified in 37 patients (49%), including Enterobacterales (n = 11), *Pseudomonas aeruginosa* (n = 10), *Streptococcus* species including *S. pneumoniae* (n = 9), *Haemophilus influenzae* (n = 8) and *Staphylococcus aureus* (n = 6). Viral mPCR was performed in 105 patients (100%). A respiratory virus was detected in 34 patients (31%), including VRS (n = 11), influenza (n = 8), rhinovirus (n = 8), para influenza (n = 3), metapneumovirus (n = 3) and coronavirus (n = 2). Two viruses were detected in one patient. Seventeen patients (16%) had a mixed infection (Fig. 1). No atypical bacteria was detected.

**Table 1:** Baseline characteristics.

Variable	Missing values	Results
Age (year), median [25–75]	0	69 [62.3–74.7]
Sex (M/W), n (%)	0	71/34 (68/32)
BMI (kg/m^2^), median [25–75]	3	25 [21–31.2]
Smoking habits, n (%)		
Non smoker	0	4 (3.8)
Former smoker	0	47 (44)
Active	0	54 (51)
FEV1 (mL), median [25–75]	45	900 [670–1230]
FEV1 (%), median [25–75]	20	37 [27–47]
FEV1/FVC (%), median [25–75]	35	46.5 [37–58]
COPD ≥ GOLD III, n (%)	20	68 (80)
No. previous AECOPD in the year, median [25–75]	26	2 [0–3]
No. previous hospital admission for AECOPD in the past year, median [25–75]	35	1 [0–2]
Bronchial bacterial colonization, n (%)	42	19 (30)
Bronchial *Pseudomonas aeruginosa* colonization, n (%)	0	13 (12)
Influenza vaccination, n (%)	54	26 (51)
Long-term treatment, n (%)		
Long-term oxygen therapy	0	36 (34)
NIV	0	22 (21)
LABA	0	76 (72)
LAMA	0	73 (69)
ICS	0	56 (53)
Systemic corticosteroid	0	5 (4.7)
Azithromycin	0	14 (13)
Comorbidities, n (%)		
Arterial hypertension	0	65 (61.9)
Ischemic cardiomyopathy	0	24 (22.8)
Arrhythmia	0	21 (20)
Diabetes	0	22 (20.9)
Chronic kidney disease	0	11 (10.4)
Cancer	0	19 (18)
ICU admission data		
Mechanical ventilation in the first 24 h, n (%)	0	96 (91.4)
Invasive ventilation	0	15 (14)
Non-invasive ventilation*	0	91 (86)
SAPS2 score, median [25–75]	1	31 [25–38]

Results are reported as median and inter-quartile range [25–75] or number and frequency.

*BMI* body mass index, *M* man, *W* woman, *FEV1* forced expiratory volume in one second, *FVC* forced vital capacity, *GOLD* global initiative for chronic obstructive lung disease, *NIV* non-invasive ventilation, *LABA* Long-Acting β2-adrenergic receptor Agonists, *LAMA* long-acting muscarinic acetylcholine receptor antagonists, *ICS* inhaled corticosteroid, *ICU* intensive care unit, *SAPS2* simplified acute physiologic score^21^.

*10 patients received first non-invasive ventilation and then invasive mechanical ventilation.

**Table 2:** Microbiological investigation.

	Test performed, n (%)	Positive result, n (%)
Bacteriological examination of respiratory tract specimen (n = 75)*	75/105 (72.3)	37/75 (48.6)**
Sputum	66/105 (63)	26/66 (39)
Tracheal aspirate	30/105 (28.5)	8/30 (27)
Urinary antigen tests		
*Legionella pneumophila*	63/105 (60)	0/63 (0)
*Streptococcus pneumoniae*	75/105 (71)	7/75 (9.3)
Blood cultures	39/105 (37)	1/39 (2.5)
Respiratory multiplex PCR (nasopharyngeal swab)	105 (100)***	34/105 (32)

*Including sputum or tracheal aspirate. Sputum and tracheal aspirate were performed in 20 patients.

**Two bacterial species were identified in 5 patients, and 3 bacterial species in one patient.

***Two viruses were identified in one patient (rhinovirus and VRS).

Baseline characteristics and symptoms were similar between patients with bacterial and non-bacterial AECOPD, except for a higher PCT level at enrolment in the bacterial AECOPD group (median of 0.21 [0.11–0.99] µg/L *vs*. 0.1 [0.07–0.18] µg/L for a threshold at 0.5 µg/L; *P* = 0.01) (Annex Table S1).

Antibiotic therapy was initiated after a median of 0 day (inter-quartile range [0–1]) in 91 patients (86.6%), more often in those with bacterial AECOPD, as compared with their counterparts (100% vs. 79%; *P* = 0.002). The overall duration of antibiotic therapy (including antibiotics administered for the episode of AECOPD and those administered for superinfection) was shorter in non-bacterial AECOPD patients (5.6 days *vs*. 9 days; *P* = 0.0006), notably in those with viral infection alone (Table 3). There was no between-group difference in any clinical outcomes (rate of hospital-acquired infection, ICU and hospital lengths of stay, mortality rate).

**Table 3:** Initial management and clinical outcomes.

	**Non-bacterial AECOPD, n = 68/105**		**Bacterial AECOPD, n=37/105 **		*P*
**Antibiotics exposure**					
**All patients**					
Antibiotic therapy, n (%)	54 (79.4)		37 (100)		0.002
Overall duration (days), mean (SD); median, n [25–75]	5.6 (3.7); 6 [2.5–7]		9 (3.9); 7 [6–12]		0.0006

**Patients with microbiological documentation**	Virus (n = 17)	No Documentation (n = 51)	Virus + Bacteria (n = 17)	Bacteria (n = 20)	
Antibiotic therapy, n (%)	15 (88.2)	39 (76.5)	17 (100)	20 (100)	
Overall duration (days), mean (SD) median, n [25–75]	5.5 (4.3), 5 [3–7]	5 (3.4), 6 [1.5–7]	8.4 (4.06), 7 [6–10]	7.7 (3.9),7 [6–9]	
**Outcomes**					
Hospital-acquired infection, n (%)	7/68 (10)		7/37 (19)		0.35
Length of stay (days), mean (SD), median [25–75]					
In-ICU	7.2 (4.6); 7 [4–10]		9.2 (7.1); 7 [5–10]		0.23
In-Hospital	15.2 (11.4); 12 [8.6–18]		17 (10.4); 14 [10–20]		0.12
Mortality, n (%)					
In-ICU	2/68 (2.9)		4/37 (10.8)		0.22
In-hospital	5/68 (7.3)		4/37 (10.8)		0.81
Post-hospital discharge*	5/63 (7.9)		4/33 (12.1)		0.81
Hospital readmission after discharge, n (%)	18/63 (28.5)		13/33 (39)		0.77

Results are reported as median and inter-quartile range [25–75] or number and frequency.

*Follow-up duration after hospital discharge (days), mean (SD), median [25–75]: 110 (117), 56 [10–199].

### Sensitivity analyses

In a first sensitivity analysis, excluding patients without complete conventional respiratory microbiological investigation (n = 30), the overall duration of antibiotic therapy differed between patients with bacterial AECOPD and those with non-bacterial AECOPD (8.03 days *vs.* 5.16 days; *P* = 0.01). In a second sensitivity analysis, excluding patients without introduction of antibiotics (n = 14, all in the non-bacterial AECOPD group), the overall duration of antibiotic therapy was no longer different (6.5 days *vs*. 8 days; *P* = 0.11) (Annex Table S2).

## Discussion

In this pilot study analysing the microbial aetiologies of severe AECOPD, we found an overall microbiological documentation rate of 50% when combining conventional tests with respiratory viral mPCR. Overall, a virus was detected in one third of cases, associated or not with bacterial species. Antibiotic therapy was shorter in the non-bacterial AECOPD patients, including those with a viral documentation alone, without adverse clinical outcomes.

Antibiotics use in AECOPD is an open controversial debate. Furthermore, AECOPD is a perfect target for antibiotic stewardship given the huge number of patients concerned, and the uncertainty about the benefit of antibiotic therapy in this context. The key unresolved issue despite decades of research on this topic, is to identify which patients truly benefit from antibiotics. Recommendations^1,3^ mostly rely on clinical criteria to guide antibiotic therapy, based on Anthonisen’s criteria. However, this strategy is not founded on robust evidence, and cannot alone guide antibiotic therapy decision in all situations, as those criteria have been initially validated in an ambulatory care setting^16^, involving less severe patients than those admitted to the ICU, and should therefore be interpreted with caution in the ICU setting.

In this context, biomarkers as CRP or PCT have been proposed as an approach to improve antibiotic usage in AECOPD. Recent studies evaluating CRP-guided strategies in the outpatient^4^ or hospital^5^ settings are encouraging. PCT-guided strategies^6–9^ are providing more heterogeneous results, and have raised concern on a potential excess of mortality in the ICU setting^9^. Consequently, routine utilisation of biomarkers is not currently recommended in AECOPD^1,3^. mPCR-guided strategy is a theoretical good candidate, providing rapid identification of a viral aetiology of AECOPD, thus allowing to avoid unnecessary antibiotics use if bacterial infection is ruled out. Some studies focussing on LRTI^10–12^ have suggested a potential interest of such molecular strategies. To our knowledge, apart from a retrospective cohort of hospitalized COPD patients^13^, there is however no study documenting the potential benefit of mPCR to guide antibiotic therapy for AECOPD patients, especially in the ICU setting. Moreover, the usefulness of the combination of rapid diagnostic testing and antimicrobial stewardship bundle should be assessed, as it has been suggested in community acquired pneumonia and other respiratory illnesses^17^.

Our findings suggest a potential benefit of viral mPCR on antibiotics streamlining in severe AECOPD. The population of our study is representative of severe COPD patients (median FEV1 of 37%) admitted for life threatening acute exacerbation (including mechanical ventilation in 90% of them), which ensures external validity to explore the risk–benefit ratio of antibiotics saving in such patients. Indeed, antibiotics use in AECOPD patients admitted to the ICU is the only setting were the rationale for antibiotic therapy is supported by a randomized controlled trial suggesting a beneficial effect on mortality^18^. Furthermore, a recent study assessing the usefulness of a PCT-guided strategy for saving antibiotics in this context have brought some concern about the safety of such a strategy^9^.

In our study, clinical data were not discriminant enough to distinguish bacterial and non-bacterial episodes of AECOPD, or to predict a viral aetiology of AECOPD. The shorter duration of antibiotics therapy in the non-bacterial AECOPD group could partly be explained as a consequence of the rapid information of a viral aetiology provided by the respiratory viral mPCR possibly combined with the absence of bacterial microorganisms. It should be acknowledged that it is the lack of a bacterial pathogen rather than the evidence of a respiratory virus that finally prompt clinicians to stop empiric antibiotic therapy. Altogether, these results highlight the contribution of the combination of the results of the conventional respiratory microbiological investigation and viral respiratory mPCR for improving the therapeutic management of severe AECOPD.

Last, this study was conducted before the pandemic, underlining the importance of the microbiological documentation of AECOPD, which should be as exhaustive as possible, in order to optimize the management of infectious aetiologies of AECOPD. This has become even more important since the pandemic, which has been associated with dramatic changes in the management of patients with chronic respiratory diseases. French nationwide studies have suggested that patients with chronic pulmonary diseases hospitalized for COVID-19 were at higher risk of developing severe COVID-19 and had higher rates of unfavourable outcomes, despite lower hospitalization rates^19,20^. The pandemic has caused considerable disruption to healthcare systems, with many changes in practices during the first waves, particularly concerning the prescription of steroids and antibiotics. Current 2023 GOLD recommendations^1^, at a distance from the pandemic, recommend that antibiotics should be used in COPD exacerbations according to the usual indications whether or not there is evidence of SARS-COV-2 infection. However, they warn that “people with COPD who develop COVID-19 are reported to more frequently develop bacterial or fungal coinfections”.

### Limitations

Limitation of the study are related to (i) the limited sample size of the population studied precluding any definite conclusion between the microbiological documentation of AECOPD and the clinical outcomes (type 2 error); (ii) a possible centre effect, since both study centres are involved in research about utility of respiratory mPCR; (iii) a significant difference in baseline PCT levels between the two groups, with a higher PCT level in the bacterial group that could have influenced the management of antibiotic therapy; and (iv) unknown confounding in the comparison of groups due to the open study design.

## Conclusion

To summarize, this exploratory study suggests a potential usefulness of respiratory viral mPCR on antibiotics stewardship during acute exacerbations of severe COPD, with reassuring data about safety. In this context, further evaluation of the therapeutic and prognostic impact of molecular tests with enlarged respiratory panels is warranted (Use of MULTIplex PCR, Procalcitonin, and Sputum Appearance to Reduce Duration of Antibiotic Therapy During Severe COPD EXAcerbation: A Controlled, Randomized, Open-label, Parallel-Group, Multicenter Trial (MULTI-EXA). ClinicalTrials.gov Identifier: NCT05280132).

### Supplementary Information

Supplementary Information.

## Data Availability

The datasets used and/or analyzed during the current study are available from the corresponding author on reasonable request.
