# Supplementary material for: Diagnostic yield of viral multiplex PCR during acute exacerbation of COPD admitted to the intensive care unit: a pilot study
Source: Sci Rep. 2024 Jan 11;14:1057. doi: 10.1038/s41598-024-51465-1 (PMC10784589; doi:10.1038/s41598-024-51465-1)
Supplement: Supplementary file 1 — Supplementary Information. [file 41598_2024_51465_MOESM1_ESM.docx]

# **SUPPLEMENTARY INFORMATION**

**Annex Table 1**. Baseline characteristics and symptoms

|  | **Missing data**  **(non-bacterial exacerbation / bacterial exacerbation)** | **Non-bacterial**  **exacerbation**  **n=68** | **Bacterial**  **exacerbation**  **n=37** | ***P*** |
| --- | --- | --- | --- | --- |
| **Demographics and history** | | | | |
| Age (year), median [25-75] | 0/0 | 68.9 [62.8 – 74.4] | 69.7 [62.4 – 75.1] | 0.96 |
| Sex (M/F), n (%) | 0/0 | 45/23 (64/34) | 26/11 (70/30) | 0.83 |
| FEV1 (%), median [25-75] |  | 39 [27 – 46.5] | 34 [27.5 – 47] | 0.56 |
| Long term oxygen therapy, n (%) | 0/0 | 24 (35.3) | 12 (32.4) | 0.94 |
| Long term NIV, n (%) | 0/0 | 17 (25.0) | 5 (13.5) | 0.26 |
| Inhaled corticosteroid, n (%) | 0/0 | 37 (54.4) | 19 (51.3) | 0.92 |
| Systemic corticosteroid, n (%) | 0/0 | 28 (41.1) | 8 (21.6) | 0.07 |
| **Baseline symptoms and Biology** | | | | |
| Household contamination, n (%) | 1/2 | 7 (10.2) | 2 (5.4) | 0.67 |
| Influenza-like syndrome, n (%) | 1/2 | 11 (16.1) | 10 (27.0) | 0.24 |
| Rhinorrhea, n (%) | 1/2 | 11 (16.2) | 7 (18.9) | 0.86 |
| Cough, n (%) | 0/2 | 38 (55.8) | 22 (59.4) | 0.64 |
| Dyspnea, n (%) | 0/2 | 64 (94.1) | 29 (78.3) | 0.14 |
| Wheezing, n (%) | 0/2 | 60 (88.2) | 30 (85.7) | 0.96 |
| Respiratory distress, n (%) | 0/2 | 57 (83.8) | 29 (78.3) | >0.9 |
| CRP (mg/L), median [25-75] | 52/24 | 22 [5.9-100] | 50 [22-109] | 0.24 |
| PCT (µg/L), median [25-75] | 41/12 | 0.1 [0.07-0.18] | 0.21 [0.11-0.99] | 0.01 |
| WBC (G/L), median [25-75] | 23/5 | 11.1 [8.7-13.5] | 12 [9.5-15.1] | 0.27 |

Results are reported as median and inter-quartile range [25–75] or number and frequency. Abbreviations. M: male; F: female; FEV1: forced expiratory volume in one second; NIV: non-invasive ventilation; WBC: white blood count.

**Annex Table 2**. Sensitivity analysis

|  | **Non-bacterial**  **exacerbation** | **Bacterial**  **exacerbation** | ***P*** |
| --- | --- | --- | --- |
| **Population with complete conventional respiratory microbiological investigation only (n=75)** | | | |
|  | n=38 | n=37 |  |
| Overall duration of antibiotics (days), mean (SD)  median, n [25-75] | 5.16 (3.44)  6 [3-7] | 8.03 (3.91)  7 [6-9] | 0.01 |
| **Population excluding patients without initiation of antibiotics (n=91)** | | | |
|  | n=54 | n=37 |  |
| Overall duration of antibiotics (days), mean (SD)  median, n [25-75] | 6.5 (2.85)  7 [5-7] | 8 (3.91)  7 [6-9] | 0.11 |

Results are reported as mean and standard deviation (SD) or median and inter-quartile range [25–75].
